# Supplementary figures and images for: Involvement of community pharmacy pharmacists in fecal immunochemical test screening without government support in Japan
Source: PLoS One. 2025 May 23;20(5):e0322879. doi: 10.1371/journal.pone.0322879 (PMC12101737; doi:10.1371/journal.pone.0322879)

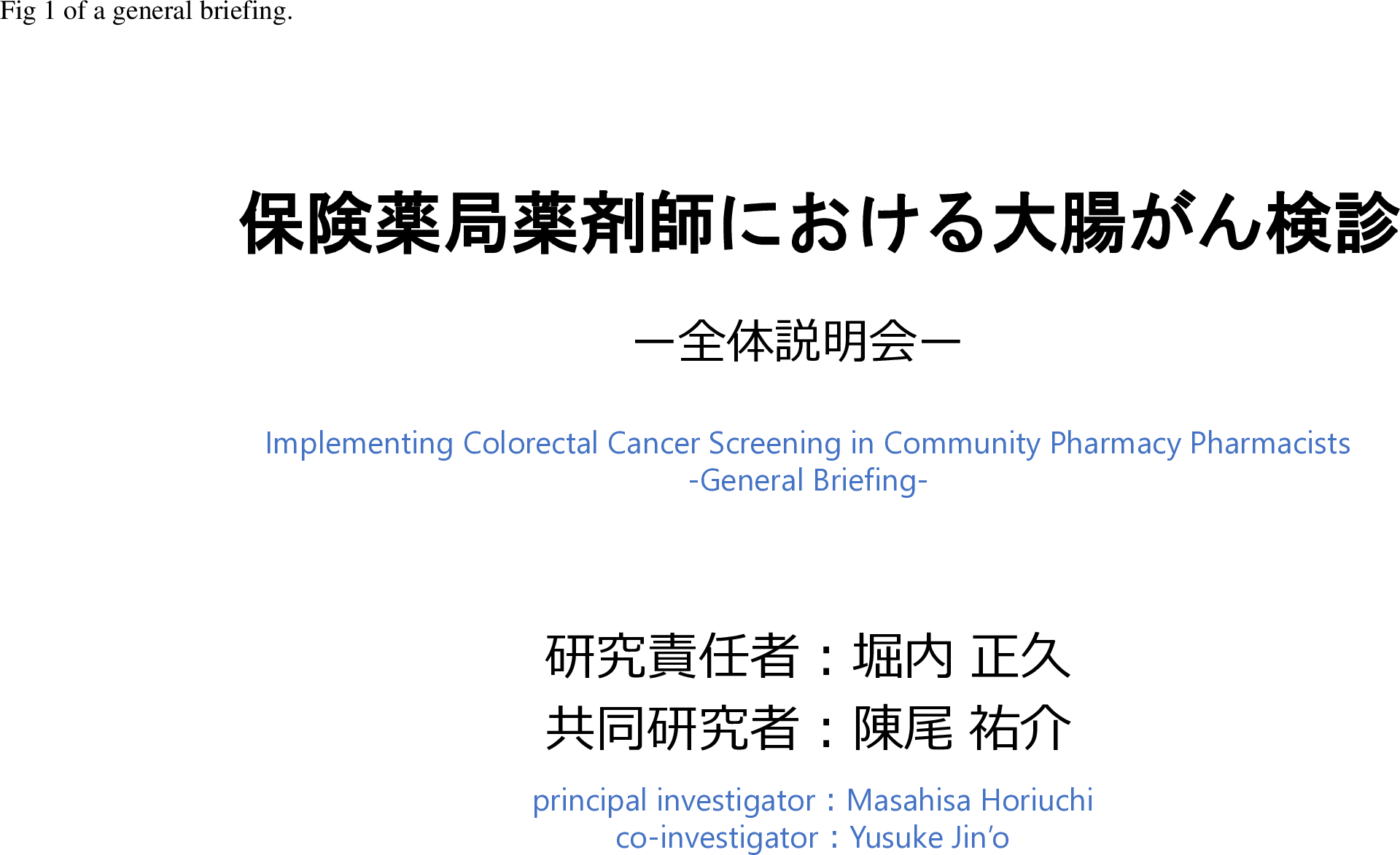

Supplement: S1 Appendix — (TIF) [file pone.0322879.s001.tif]

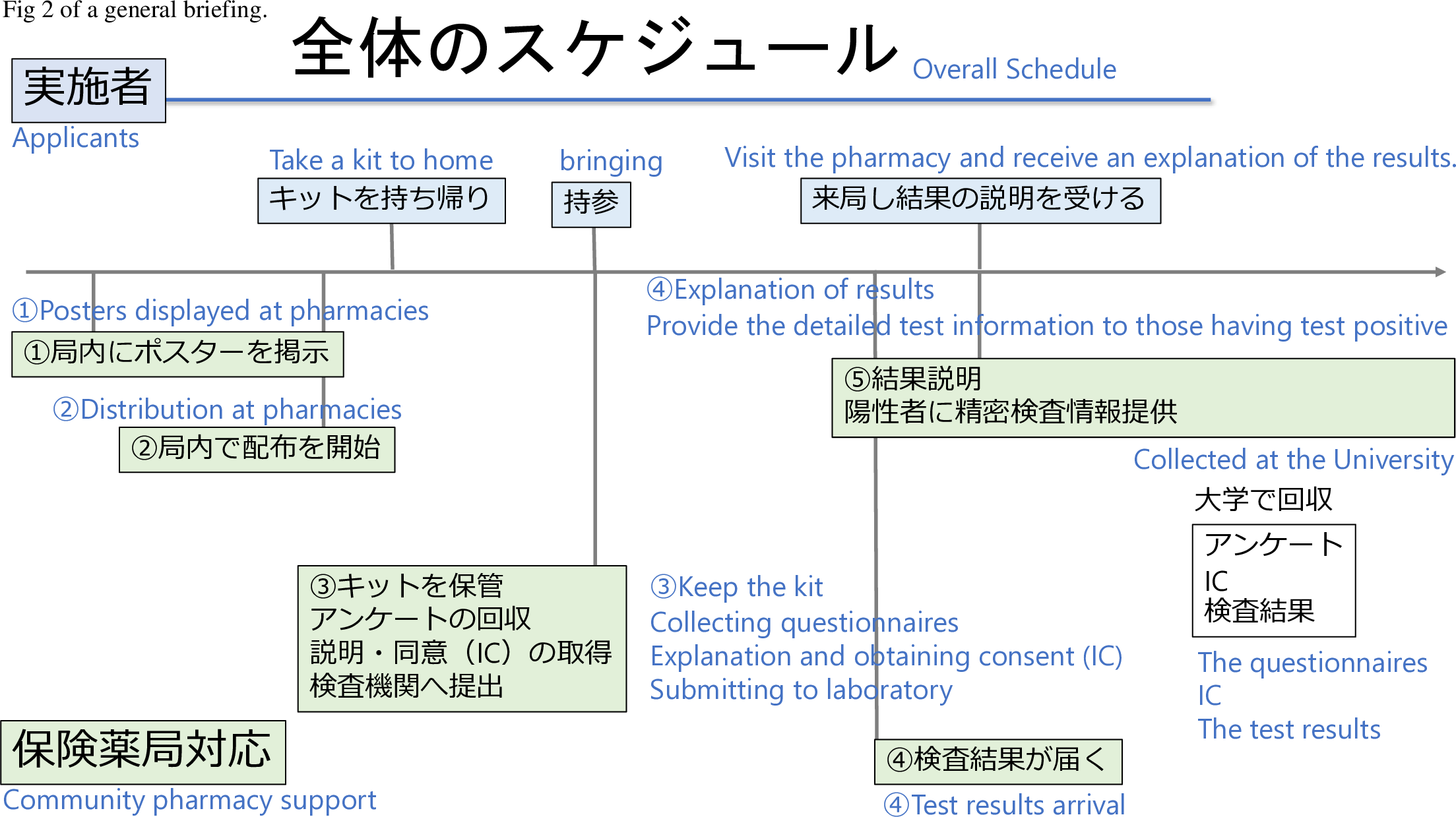

Supplement: S2 Appendix — (TIF) [file pone.0322879.s002.tif]

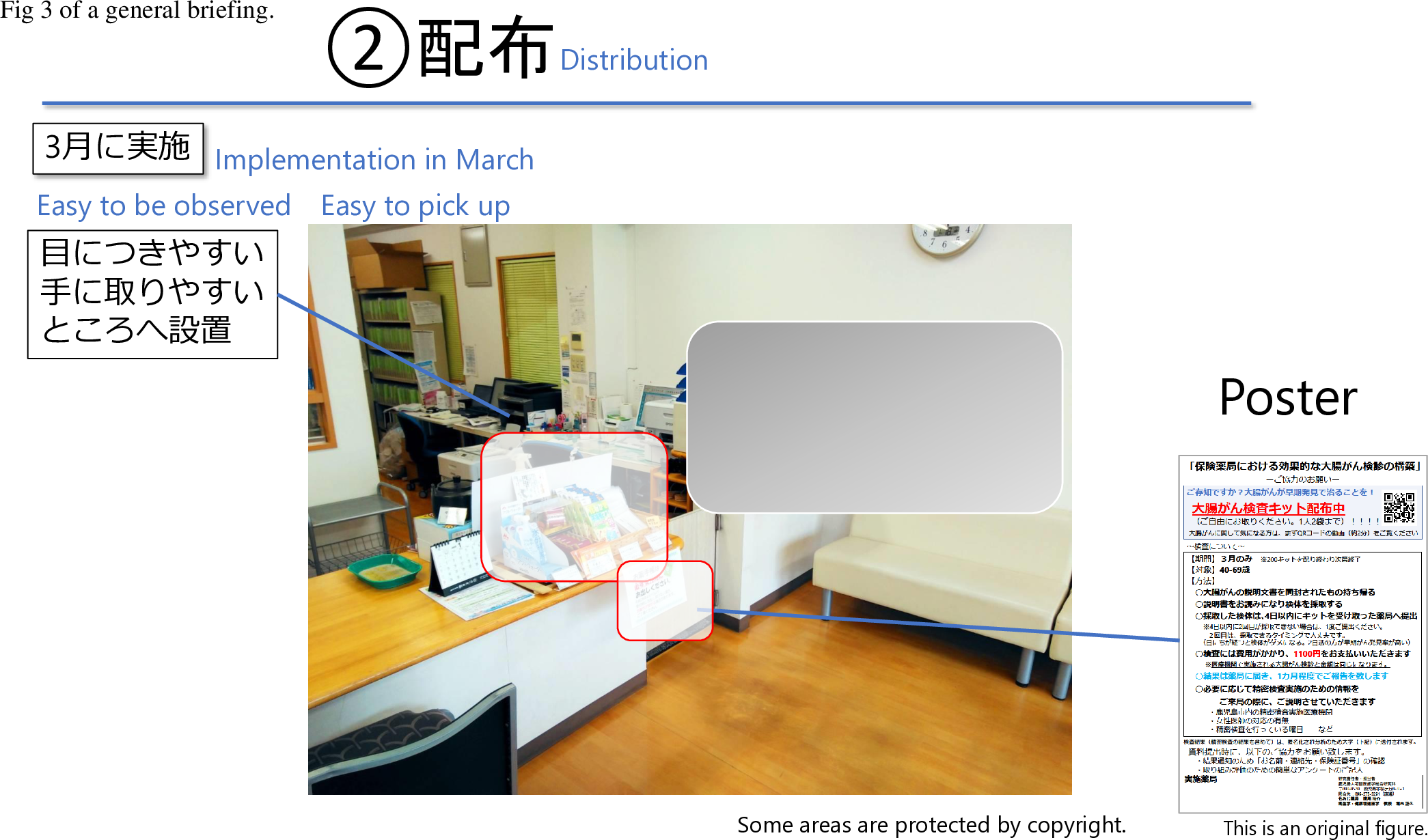

Supplement: S3 Appendix — (TIF) [file pone.0322879.s003.tif]

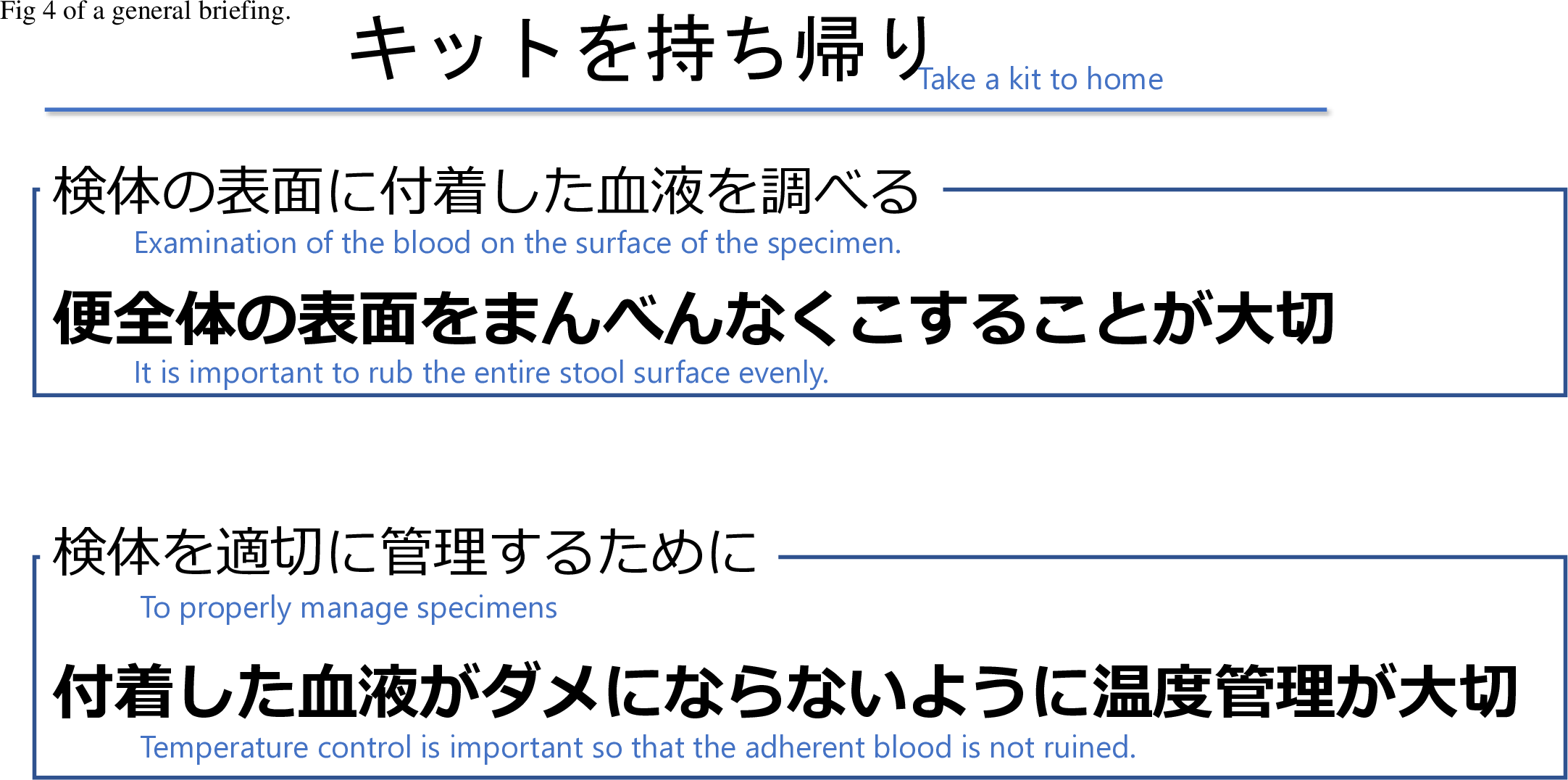

Supplement: S4 Appendix — (TIF) [file pone.0322879.s004.tif]

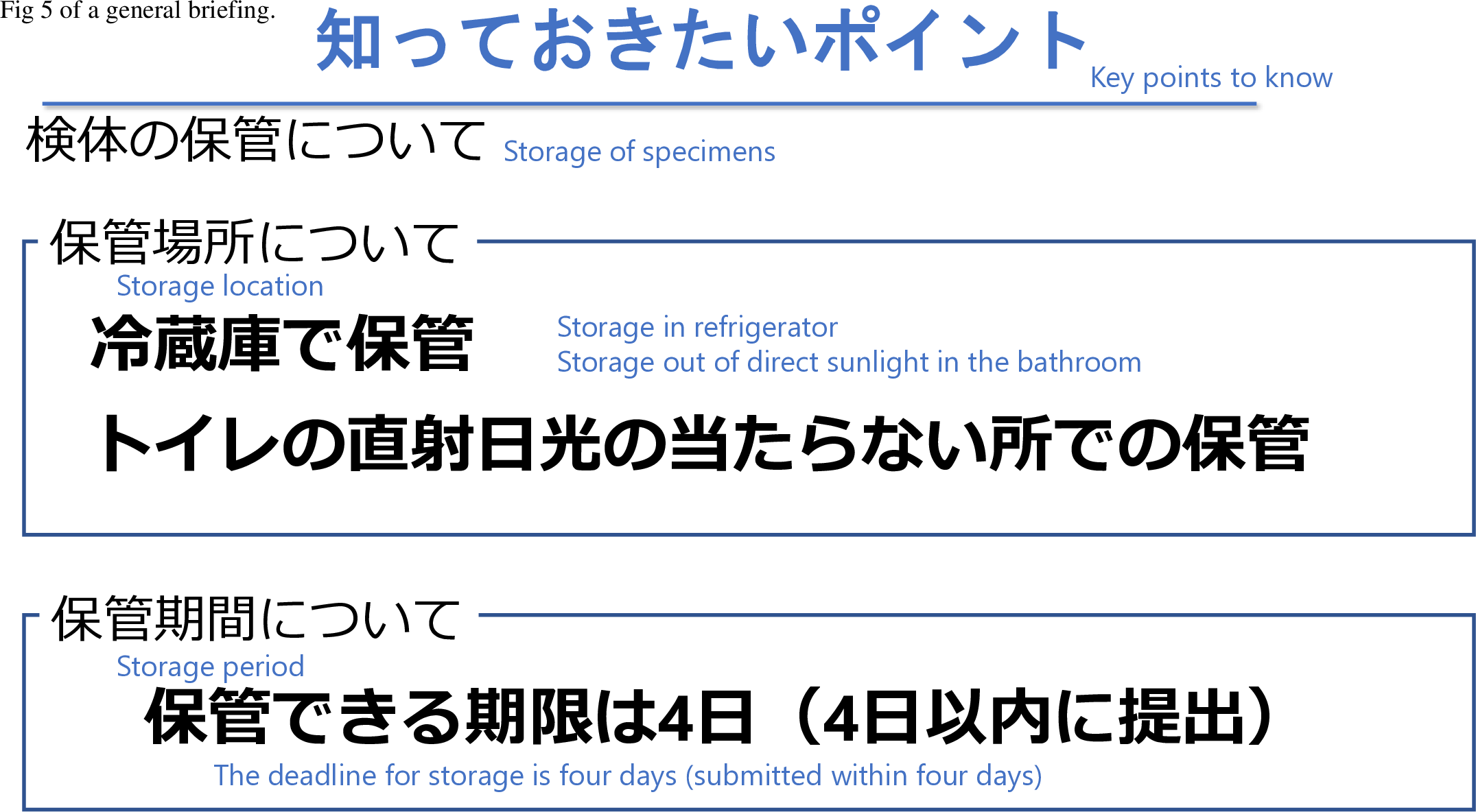

Supplement: S5 Appendix — (TIF) [file pone.0322879.s005.tif]

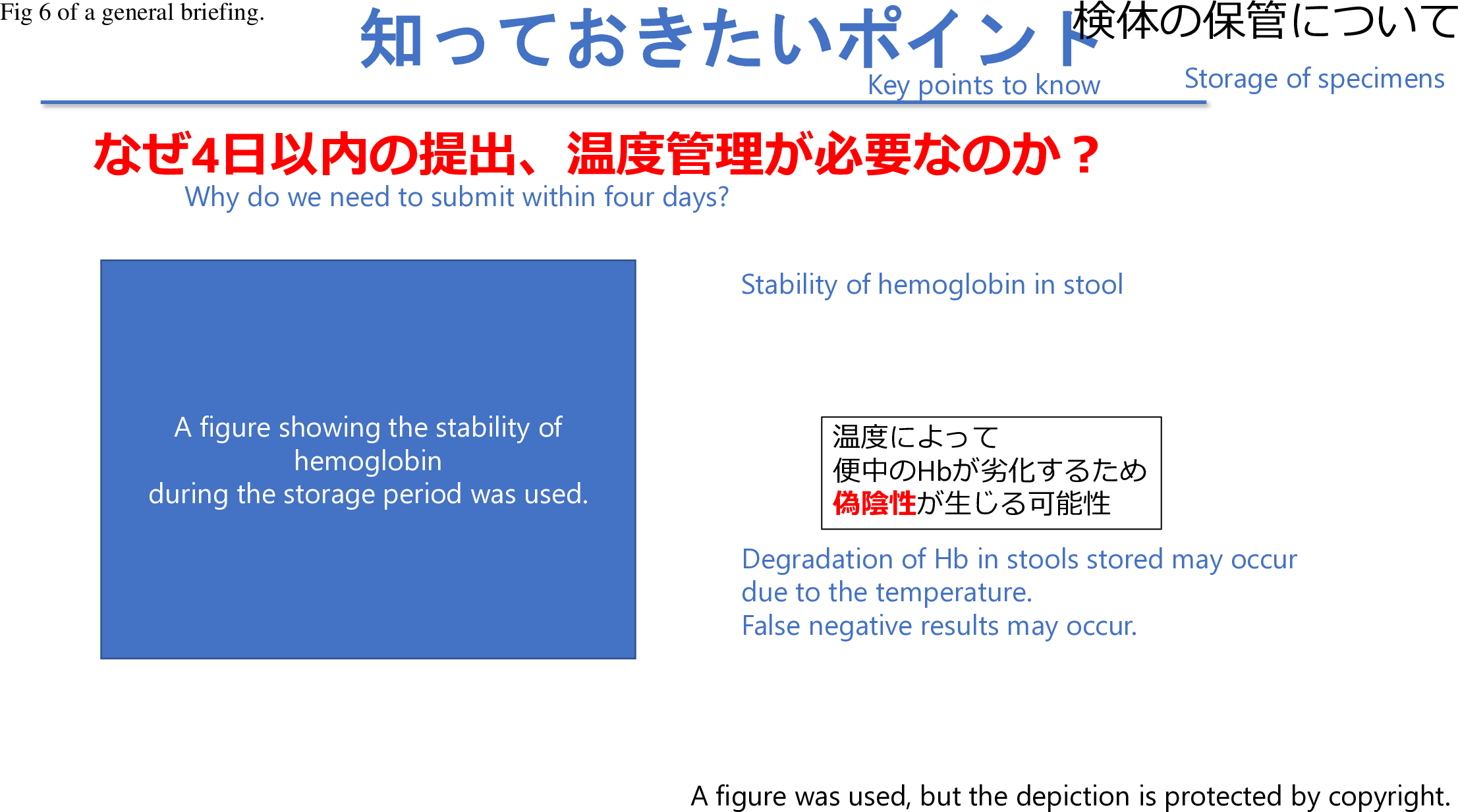

Supplement: S6 Appendix — (TIF) [file pone.0322879.s006.tif]

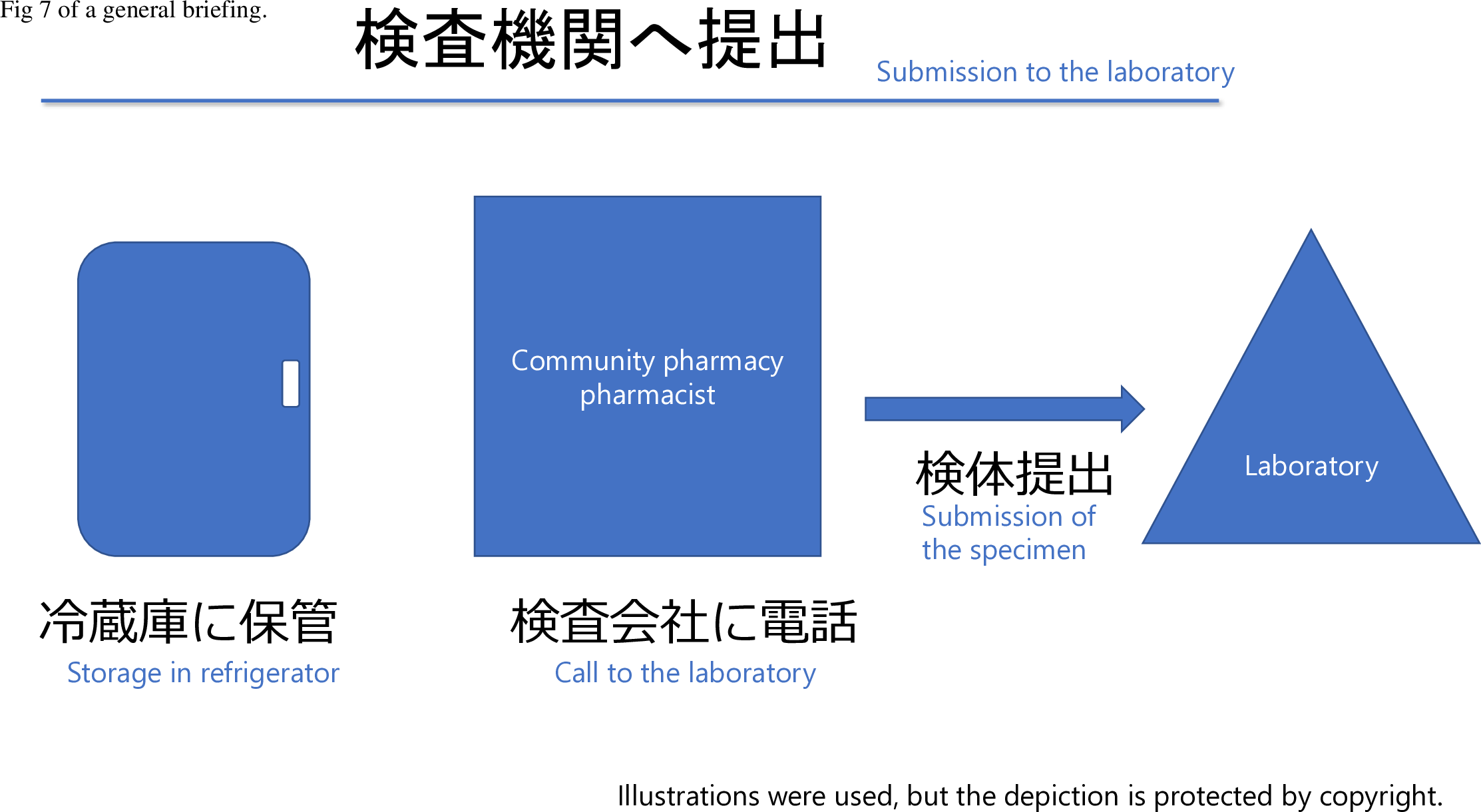

Supplement: S7 Appendix — (TIF) [file pone.0322879.s007.tif]

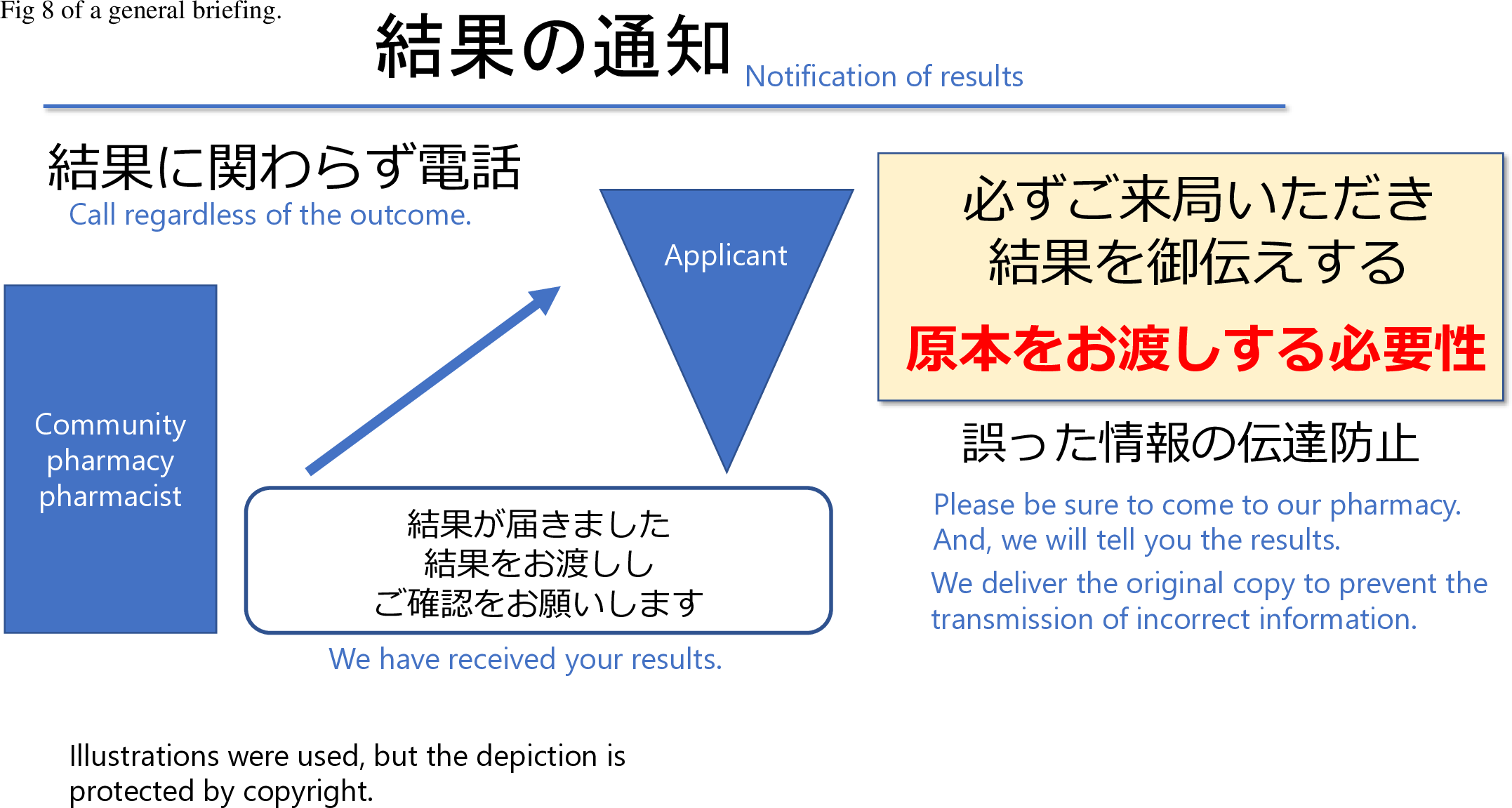

Supplement: S8 Appendix — (TIF) [file pone.0322879.s008.tif]

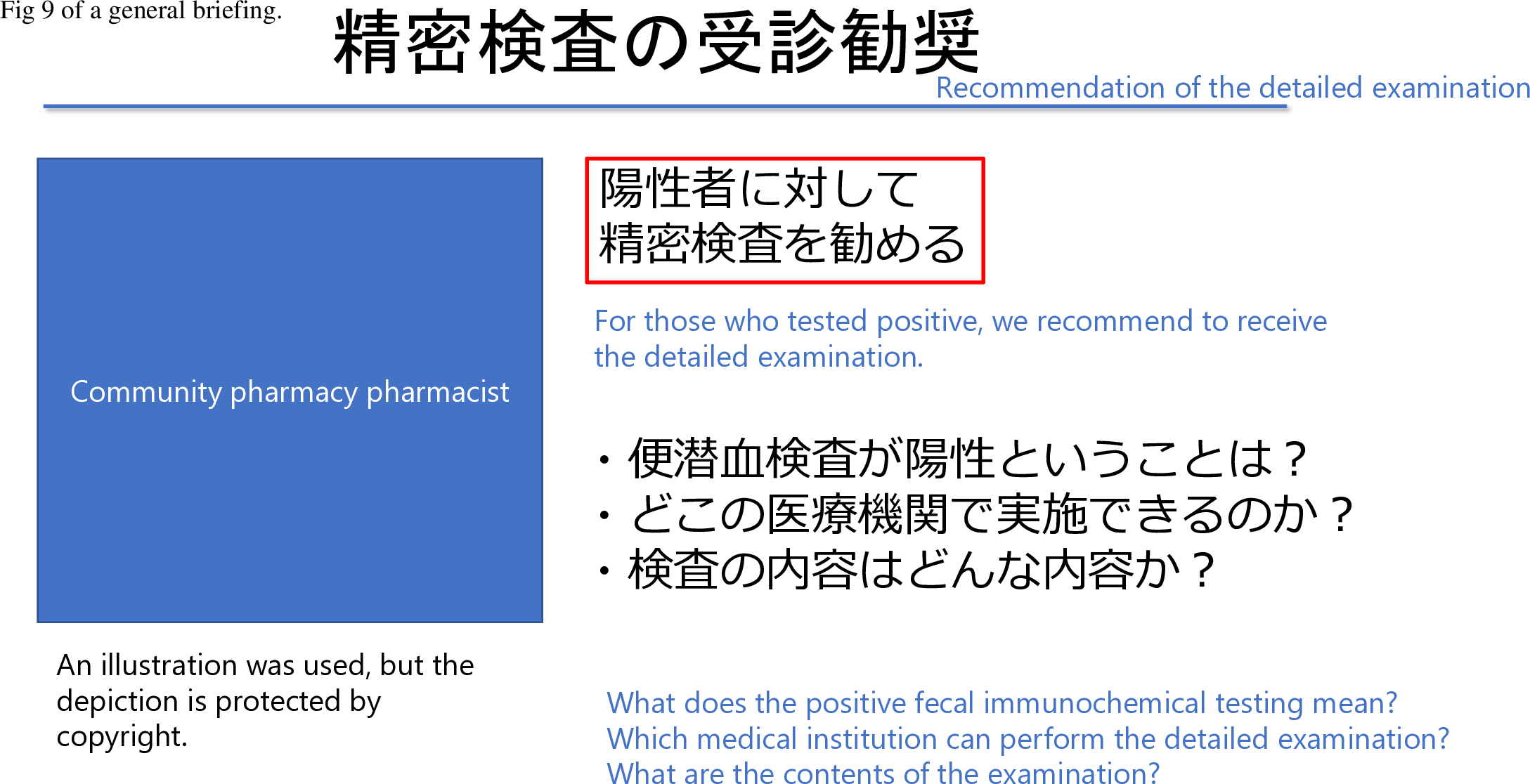

Supplement: S9Appendix — (TIF) [file pone.0322879.s009.tif]
