# Supplementary material for: Involvement of community pharmacy pharmacists in fecal immunochemical test screening without government support in Japan
Source: PLoS One. 2025 May 23;20(5):e0322879. doi: 10.1371/journal.pone.0322879 (PMC12101737; doi:10.1371/journal.pone.0322879)
Supplement: Attachment — Submitted filename: PONE-D-24-43344-review comment.docx [file pone.0322879.s010.docx]

Review comments

1. Introduction needs to be reorganized (paragraph 1; burned and incidence including Japa, 2: uptake and screening, 3: health promotion, 4; the Community pharmacy pharmacists and study aim), need to be consistence, if we say uptake should be uptake throughout, if screening rate should have to the same throughout, there is a need for rephrasing and rearrangement of the paragraphs.
2. Introduction 49 to 51; this has to be part of second paragraph
3. Introduction line 52; this sound to be part of paragraph one which speak on burden and has to include the incidence as well.
4. Introduction line 66 to 67; is not clear what is six-years system mean and why is so important for this study.
5. Methodology Participants: line 81 to 90; you have to explain the characteristics of participants/ or participating pharmacies, and including their exclusion criteria but I see also you included the part of the results in this section.
6. Methodology is missing the study design part, is it cross-sectional, survey, or randomized study???
7. Methodology Examinations line 92; this has to be rephrased to study procedure
8. Methodology line 93 to 95; the authors need to mention the sensitivity cut point you are referring to is it 98%, 35% etc and explain the usually CRC screening Methode used in Japan. And you need to start this procedure part by explaining what was done, how, when and where, and you mention the kits used for this and its sensitivity.
9. Methodology data line 100 this has to be data collection method
10. Methodology data line 106 to 109; the authors need to stick to CRC and not COVID-19, there is
11. Result Correlation line 131; why did we do the correlation; do we have correlation procedure in analysis like Kappa.
12. Result Table 1: need to be the table for participants characteristics, age., sex, how many tested (tested negative and tested positive), insurance (mention only the insurance with significance number, and the other with lower number should be grouped under one part mentioned as other insurance). The one you provide fits to be part of supplementary tables.
13. Result line 140 to 143; this is parts for participant in methodology and not results; Authors need to remove the results in this section and include it on respective section
14. Result line 156 to 158; it has not explained well in introduction even on the methodology and statistical part if it is needed it needs to be reflected on the study aim on introduction and well as in analysis procedure.
15. Discussion of this study is not clear and straightforward, the authors explained all results as in results section, some are not in the scope of this study, in general needs to be focused.
16. Discussion; the first paragraph needs to have your 3 or 4 important findings according to your aim. Thereafter, the following paragraphs has to contain the detailed information of each mentioned important findings from 1^st^ paragraph, you compare and contrast against evidence from previously.
17. Discussion line 219 to 223; no need for this information in this part of discussion advised to be deleted.
18. Discussion Line 245 to 247; need to be consistence on wording choose either pharmacist-mediated CRT test or community pharmacy pharmacists, check also other part of manuscript where you mixed up these words.
19. Discussion line 249; no need to refer figure, you already mentioned in results
20. Discussion line 248 to264; this paragraph has the important finding according to the study aim the uptake following the intervention, you need to explain in detains on the uptake, comparing with other findings from other studies, and give the impression for it. The cost and profit need to be in a separate paragraph, and include in the study procedure the information on how you assessed the cost and profit.
21. Discussion line 265 to 275; need to be a separate paragraph, no need to refer table at this point already is in the result. Rephrase and mention only the important finding of which I see the uptake of community pharmacy pharmacist test CRC test by young age. Remove the other part which has no information on the uptake and the paragraph should be before the const information. And discuss in details on your result about young age are these also seen to other studies from different area or not, and if possible, why.
22. Discussion line 275 to 294; The positivity rate increase with age is important information but has not discussed well and is not clear if you’re reporting this as a limitation or you are reporting the new findings, need to separate this paragraph part of it is not scope of this study.
23. Discussion line 295 to 296; is a good finding but what are the other two you mean needs more discussion on this finding.
24. Discussion line 319 to 320; reporting the cost why it is a limitation to this study?
