# Supplementary material for: Involvement of community pharmacy pharmacists in fecal immunochemical test screening without government support in Japan
Source: PLoS One. 2025 May 23;20(5):e0322879. doi: 10.1371/journal.pone.0322879 (PMC12101737; doi:10.1371/journal.pone.0322879)
Supplement: Attachment — Submitted filename: Response to Reviewers.docx [file pone.0322879.s011.docx]

Sameen Abbas, Ph.D.

Academic Editor

PLOS ONE

Dear Prof. Abbas,

We are pleased to learn that our manuscript (PONE-D-24-43344, Involvement of the

community pharmacy pharmacists in fecal immunochemical screening without government

supports in Japan) can be considered for publication in your journal.

First of all, we explained the correction to the Table 2. We have noticed the error in in Table 2 regarding the applicants aged 50-59 in the hospital/clinic-mediated procedure. According to the correction, we have corrected the statistical results, which are marked in red. The percentage of positive results in the hospital/clinic-mediated procedure was changed from 5.8 to 5.7. In addition, we have added the data on the number of age groups (40s, 50s, and 60s) in the hospital/clinic- and health examination institution-mediated procedures in Table 3 to respond to the reviewers’ comments. The correction and addition would be helpful for a better understanding of the presentation of our manuscript.

We have responded to the reviewers’ comments point by point as follows. We have highlighted in red the sentences that have been added to the text.

**Reviewer #1:**

**1. Introduction needs to be reorganized (paragraph 1; burned and incidence including Japan, 2: uptake and screening, 3: health promotion, 4; the Community pharmacy pharmacists and study aim), need to be consistence, if we say uptake should be uptake throughout, if screening rate should have to the same throughout, there is a need for rephrasing and rearrangement of the paragraphs.**

We agreed with the comments. We have reorganized the introduction section as suggested by the queries 2-3 as below.

The screening rate is used consistently.

**2. Introduction 49 to 51; this has to be part of second paragraph**

We have moved this part to the second paragraph.

**3. Introduction line 52; this sound to be part of paragraph one which speak on burden and has to include the incidence as well.**

We have moved this part to the first paragraph.

**4. Introduction line 66 to 67; is not clear what is six-years system mean and why is so important for this study.**

The change from a four-year system to a six-year system has increased the increased content on prevention and health in addition to clinical training. Therefore, the change to the six-year system is important for this study. Accordingly, we changed the sentence (P4 L70-72 in the Manuscript).

However, pharmacists in Japan are undergoing a shift to a six-year system at university and the expansion of the educational content related to prevention and health in addition to clinical training [16].

**5. Methodology Participants: line 81 to 90; you have to explain the characteristics of participants/ or participating pharmacies, and including their exclusion criteria but I see also you included the part of the results in this section.**

We have removed the results part of this section. We have added the information to the sentence (P5 L84-85 in the Manuscript).

Thirty-seven community pharmacies in Kagoshima City voluntarily participated in the study (Table 1).

**6. Methodology is missing the study design part, is it cross-sectional, survey, or randomized study???**

We have added the part in this section as Study design and participants. We added the sentence (P5 L84 in the Manuscript).

This study was conducted as a survey.

**7. Methodology Examinations line 92; this has to be rephrased to study procedure**

We have combined the sections (Participants and Examinations) and rephrased them as Study design and procedure.

**8. Methodology line 93 to 95; the authors need to mention the sensitivity cut point you are referring to is it 98%, 35% etc and explain the usually CRC screening Method used in Japan. And you need to start this procedure part by explaining what was done, how, when and where, and you mention the kits used for this and its sensitivity.**

The 2-day method used in the study is the usual CRC screening method in Japan.

We modified the sentence as follows (P5 L94-96 in the Manuscript).

Considering the sensitivity of the test, a 2-day method was used, which is the usual method for CRC screening in Japan [20].

As suggested, we explained what was done, how, when, and where in the study design and procedure section (P5 L86-93 in the Manuscript).

Kits were distributed by participating pharmacies between March and the first week of April 2023 (Fig 1). Stool samples were collected at home, brought to a community pharmacy, and sent to the laboratory (Clinical Pathology Laboratory Co. Ltd., Kagoshima, Japan). The collection period was from March to August 31, 2023. The results were reported to the community pharmacist in charge and then to the participants. For patients who tested positive, explanations were provided about the medical institutions that performed detailed examinations.

We have added the sentences (P5-6 L96-98 in the Manuscript).

According to the manufacturer, the cut-off value is 100 ng Hb/ml. Therefore, the sensitivity of the 2-day method for CRC is 100.0% (74.1-100.0% as 95% confidential intervals) [20].

**9. Methodology data line 100 this has to be data collection method.**

We have rephrased it as suggested.

**10. Methodology data line 106 to 109; the authors need to stick to CRC and not COVID-19.**

We have explained the sentences. These sentences are the reason for selecting the data.

We have added the sentence and removed the sentence for the redundancy (P7 L120-122 in the Manuscript).

The time of the data was chosen because the COVID-19 pandemic could affect the screening rate of general health examinations, including CRC screening.

**11. Result Correlation line 131; why did we do the correlation; do we have correlation procedure in analysis like Kappa.**

As we understand it, the kappa statistic is used to assess the reproducibility using the same samples. Since the participants in each pharmacy were different, we analyzed the correlation between the number of pharmacists in the pharmacy and the number of kits distributed in the pharmacy.

**12. Result Table 1: need to be the table for participants characteristics, age., sex, how many tested (tested negative and tested positive), insurance (mention only the insurance with significance number, and the other with lower number should be grouped under one part mentioned as other insurance). The one you provide fits to be part of supplementary tables.**

We are sorry that we did not receive information about the participants who did not take FIT. Therefore, we cannot prepare the table as suggested. We have corrected the ethical sentence (P8 L135-136 in the Manuscript).

Informed consent was obtained from all participants with FIT examination before they completed the questionnaire.

**13. Result line 140 to 143; this is parts for participant in methodology and not results; Authors need to remove the results in this section and include it on respective section**

We have moved it to the methodology section.

**14. Result line 156 to 158; it has not explained well in introduction even on the methodology and statistical part if it is needed it needs to be reflected on the study aim on introduction and well as in analysis procedure.**

We have added the explanations in the Introduction section (P3 L57-58 in the Manuscript).

In Japan, there are two FIT procedures that are provided by hospitals/clinics and health examination institutes.

**15. Discussion of this study is not clear and straightforward, the authors explained all results as in results section, some are not in the scope of this study, in general needs to be focused.**

We agree with the proposal. We have attempted to rewrite the discussion section as indicated below.

**16. Discussion; the first paragraph needs to have your 3 or 4 important findings according to your aim. Thereafter, the following paragraphs has to contain the detailed information of each mentioned important findings from 1st paragraph, you compare and contrast against evidence from previously.**

We described the key points of the study (P18 L229-236 in the Manuscript).

CPP-mediated CRC screening was conducted in Japan. More than one-third of the FIT participants with FIT were not taking any medication at the pharmacy where they were tested. Compared with conventional CRC screening procedures, a higher percentage of the younger generation was screened, but the rate of detailed examination was lower. The 2-day method was used 100% of the time. In addition, the rate of pathological findings on the detailed examination in the CPP-mediated CRC screening was similar to that in the hospital/clinic and health examination-mediated procedures. These results suggest that the CPP-mediated procedure could be used as a novel screening procedure for CRC.

**17. Discussion line 219 to 223; no need for this information in this part of discussion advised to be deleted.**

We have removed the sentences.

**18. Discussion Line 245 to 247; need to be consistence on wording choose either pharmacist-mediated CRT test or community pharmacy pharmacists, check also other part of manuscript where you mixed up these words.**

We have used the term, community pharmacy pharmacists as CPPs, consistently throughout the text.

**19. Discussion line 249; no need to refer figure, you already mentioned in results**

We have deleted the information.

**20. Discussion line 248 to264; this paragraph has the important finding according to the study aim the uptake following the intervention, you need to explain in detains on the uptake, comparing with other findings from other studies, and give the impression for it. The cost and profit need to be in a separate paragraph, and include in the study procedure the information on how you assessed the cost and profit.**

We appreciated the suggestion. We have detailed the comparison of the implementation rate in Spain (P19-20 L262-267 in the Manuscript).

Spain was based on the number of people tested per number of recommendations in letters, while the current study was based on the number of people tested per number of kits distributed, so there is no easy comparison, but the current study may not have been more convenient due to the limited number of participating pharmacies, and the costs associated with the test were charged, resulting in a lower implementation rate.

We separated the paragraph in the discussion section, and added the information on the cost of the procedures (hospital/clinic-mediated procedure and health examination institution-mediated procedure) in the study procedure section (P6 L103-105 in the Manuscript).

According to the local government rule, the other two procedures (hospital/clinic-mediated procedure and health examination institution-mediated procedure) are performed by the cost of self-payment, 600 JPY and 1100 JPY, respectively.

**21. Discussion line 265 to 275; need to be a separate paragraph, no need to refer table at this point already is in the result. Rephrase and mention only the important finding of which I see the uptake of community pharmacy pharmacist test CRC test by young age. Remove the other part which has no information on the uptake and the paragraph should be before the const information. And discuss in details on your result about young age are these also seen to other studies from different area or not, and if possible, why.**

We have removed the part that does not provide information on the screening rate as suggested. We have separated the paragraph related to the sensitivity of the test.

We discussed in detail the results of the higher screening rate at younger ages in the CPP-mediated procedure. We have added the following sentences (P21 L285-291 in the Manuscript).

A relatively large number of people were enrolled in the Association Health Insurance Plan. Therefore, employees of small and medium-sized enterprises who had not yet undergone CRC screening were able to take the test. Women of working age who visited the pharmacy with their children or parents may have been screened. These suggest that the pharmacies were in the community and provided an environment conducive to screening, then the applicants in the CPP-mediated procedure were relatively young.

**22. Discussion line 275 to 294; The positivity rate increase with age is important information but has not discussed well and is not clear if you’re reporting this as a limitation or you are reporting the new findings, need to separate this paragraph part of it is not scope of this study.**

We agree with the suggestion and have removed this part.

**23. Discussion line 295 to 296; is a good finding but what are the other two you mean needs more discussion on this finding.**

We analyzed the people who received the detailed examinations in the hospital/clinic- procedure and in the health examination institution-mediated procedures. Fewer people in their 40s and 50s received a detailed examination in the health examination institution-mediated procedure compared with people in their 60s. The relatively high proportion of young people among those with a positive FIT is one of the reasons for the low rate of detailed examinations in the CPP- mediated procedure.

We have added the data to Table 3, and we added the following sentences in the Discussion section (P22 L311-316 in the Manuscript).

The people who received the detailed examinations in the CPP-mediated procedure were relatively young (40-59 years: 46.7%, Table 3). Fewer people in their 40s and 50s than in their 60s received a detailed examination in the health examination institution mediated procedure. The relatively high proportion of young people among those with a positive FIT may be one of the reasons for the low rate of detailed examinations in the CPP-mediated procedure.

**24. Discussion line 319 to 320; reporting the cost why it is a limitation to this study?**

The cost verification in this study was done with the fewest variables. We thought that more careful verification was needed, so the cost calculation is raised as a limitation. We have modified the sentences according to the concept (P23 L337-340 in the Manuscript).

Second, this study reported the cost of implementing FIT. Verification of cost-effectiveness was required in several places. In this study, the verification was done with the fewest variables. More valuable evidence was needed to determine whether the CPP-mediated procedure can take root in society [36].

**Reviewer #2: Thank you for the manuscript. I have a few questions regarding its content:**

**Abstract:**

**1. Could key statistical findings be added to better highlight the study’s impact?**

We appreciate the suggestion. We have added the main statistical results in the section.

We have added the following sentences in the Abstract section as follows (P2 L31-36 in the Manuscript).

A significantly higher percentage of people in their 40s underwent the test in the CPP-mediated procedures (35.2% vs. 14.3% in the hospital/clinic, and 21.8% in the health examination institution, respectively, *p* < 0.01). The percentage of participants who underwent a detailed examination was significantly lower than in the other two groups (46.7% vs. 87.3% in the hospital/clinic, and 83.0% in the health examination institution, respectively, *p* < 0.05).

**2. How does the community pharmacy approach specifically address gaps in CRC screening participation compared to other methods?**

We appreciate the suggestion. We have added the sentences (P3 L40-42 in the Manuscript).

In particular, the CPP-mediated procedure may address gaps by increasing the number of young people and people with low opportunity to receive CRC screening.

**Methods:**

**3. Was there variability in how pharmacists communicated the procedure, and could this variability have influenced participant uptake?**

Yes, there was. The degree to which pharmacists communicated the procedure may have influenced participants' uptake. We have added these sentences to the study limitation in the Discussion section (P24 L343-346 in the Manuscript).

Although the pharmacists volunteered to participate in this implementation, there was variability in how they communicated the procedure. This variability may have influenced the actual CRC screening rate.

**4. Were standardized protocols in place to ensure proper storage and transport of the returned FIT kits?**

Yes, they were. The standardized protocols were used. We explained the standardized protocols to the participating pharmacists in the general briefing prior to the implementation. The handout will be submitted as the supplementary material with additional explanations in English. We have added the sentence in the Study design and procedure (P5 L85-86 in the Manuscript).

The participating pharmacists were trained in a general briefing (supplementary material) before the implementation.

**Results:**

**5. Why was the uptake rate lower compared to international benchmarks? Could cultural or logistical factors have played a role?**

We appreciated the suggestion, which was similar to Reviewer 1's 20th question of Reviewer 1. We have added the following sentences to the Discussion section (P19-20 L262-267 in the Manuscript).

Spain was based on the number of people tested per number of recommendations in letters, while the current study was based on the number of people tested per number of kits distributed, so there is no easy comparison, but the current study may not have been more convenient due to the limited number of participating pharmacies, and the costs associated with the test were charged, resulting in a lower implementation rate.

**6. What could explain the significantly lower rate of undergoing detailed examinations in the pharmacy group?**

We analyzed the people who received the detailed examinations in the hospital/clinic and health examination institution procedures. The data were added to Table 3. The people who received the detailed examinations in the CPP-mediated procedure were relatively young (40-59 years: 46.7%). We have added the data in Table 3, and we have added the following sentences in the Discussion section as follows (P22 L311-316 in the Manuscript).

The people who received the detailed examinations in the CPP-mediated procedure were relatively young (40-59 years: 46.7%, Table 3). Fewer people in their 40s and 50s than in their 60s received a detailed examination in the health examination institution-mediated procedure. The relatively high proportion of young people among those with a positive FIT may be one of the reasons for the low rate of detailed examinations in the CPP-mediated procedure.

In the Results section, we added the sentences (P15 L209-213 in the Manuscript).

The proportions of patients who underwent a detailed examination in their 40s, 50s and 60s of age were not significantly different in the CPP and hospital/clinic institution groups, respectively. On the other hand, the proportions of patients who underwent a detailed examination in their 40s, 50s and 60s were significantly different in the health examination institution group.

**Discussion:**

**7. How might this model help to address colorectal cancer screening gaps in rural or underserved populations?**

We think how to help to address CRC screening gaps as follows. In rural areas or underserved populations such as employees in small- and medium-sized companies, this model may also be available, as it has been shown that pharmacies with few pharmacists can provide this service.

**8. What strategies could be implemented to improve participation among men and individuals with lower health awareness?**

We have added the sentences in conclusion section (P24 L354-356 in the Manuscript).

This model should be widely known, even among people with lower health awareness. Posters and brochures about this CPP-mediated procedure should be prepared for the visitors to the pharmacies and public events.

**Limitations:**

**9. Could the short distribution period have influenced the results, and how could this issue be mitigated in future studies?**

Yes, it could. We have added the sentences in the limitation section (P23 L332-337 in the Manuscript).

The short distribution period could have affected the results. However, this time the procedure was conducted at the end of the fiscal year (March), in anticipation of those who had not yet been screened, as regular cancer screening begins in April in Japan. In the future, it could be conducted throughout the year, with the importance of serving as a contact point for those who have not yet undergone regular cancer screening, and especially with a reinforcement month at the end of the fiscal year.
