# Supplementary material for: Involvement of community pharmacy pharmacists in fecal immunochemical test screening without government support in Japan
Source: PLoS One. 2025 May 23;20(5):e0322879. doi: 10.1371/journal.pone.0322879 (PMC12101737; doi:10.1371/journal.pone.0322879)
Supplement: Attachment — Submitted filename: Response_to_Reviewers 20250326.docx [file pone.0322879.s012.docx]

Sameen Abbas, Ph.D.

Academic Editor

PLOS ONE

Dear Prof. Abbas,

We are pleased to learn that our manuscript (PONE-D-24-43344_R2, Involvement of community pharmacy pharmacists in fecal immunochemical screening without government

support in Japan) can be considered for publication in your journal.

We have responded to the reviewers’ comments point by point below. We have highlighted the sentences that have been added to the text in red.

**Reviewer #1:**

**1. Methodology: Minor additional suggestion on ethics. Would suggest to remove line 135 to 136: Informed consent was obtained from all participants with FIT examination before they completed the questionnaire; sounds like repetition and remain with line 137 which cover both FIT examination and enrollment.**

Thank you for your suggestion. As suggested, we have deleted the sentence.

**Reviewer #3:**

**1. The grammar needs to be improved throughout the manuscript. The language is not good.**

We have asked a native speaker, through an editing company, to proofread the manuscript. We hope this has improved the language and grammar of the manuscript. We have also included the English editing certificate for your reference.

**2. The main message of the paper is not clear. What's the main takeaway of the paper that the reader should have.**

We appreciate your question. The main message of the paper is the possible contribution that CPPs can make towards CRC prevention through FIT. This has been added in Conclusions section.

**3.** **I was confused when I was reading the paper about the intent of the authors. Are they describing how community pharmacies don't have enough uptake? They conclude that the procedure can be sustained without financial incentives, but where do authors state this in the paper?**

We appreciate your comment. Accordingly, we have added the following sentences to the Conclusions section to present the relevant information (L349-352 in the manuscript):

The uptake rate in the current study was low. However, as the procedure requires the recipient to bear the cost, increasing the uptake rate could help to financially sustain this procedure. In addition, motivation for the pharmacists themselves to engage in the activity is likely to exist, as it increases the social value of CPPs.

**Journal Requirements:**

**Please review your reference list to ensure that it is complete and correct. If you have cited papers that have been retracted, please include the rationale for doing so in the manuscript text, or remove these references and replace them with relevant current references.**

We have confirmed that there are no retracted papers cited in the manuscript. We have updated the information in ref. 31 as below.

Japanese. doi: 10.7143/jhep.45.366
